# Mutations in *CG8878*, a Novel Putative Protein Kinase, Enhance *P* Element Dependent Silencing (PDS) and Position Effect Variegation (PEV) in *Drosophila melanogaster*

**DOI:** 10.1371/journal.pone.0071695

**Published:** 2014-03-10

**Authors:** Allen McCracken, John Locke

**Affiliations:** Department of Biological Sciences, University of Alberta, Edmonton, Alberta, Canada; University College London, United Kingdom

## Abstract

Genes in multicellular organisms are expressed as part of a developmental program that is largely dependent on self-perpetuating higher-order chromatin states. The mechanism of establishing and maintaining these epigenetic events is well studied in *Drosophila*. The first known example of an epigenetic effect was that of (PEV) in *Drosophila*, which has been shown to be due to gene silencing via heterochromatin formation. We are investigating a process similar to Position Effect Variegation (PEV) using a mini-*w* transgene, called *Pci*, inserted in the upstream regulatory region of *ci*. The mini-*white* ^+^ transgene in *Pci* is expressed throughout the adult eye; however, when other *P* or *KP* elements are present, a variegated eye phenotype results indicating random *w* ^+^ silencing during development. This *P* element dependent silencing (PDS) can be modified by the haplo-suppressors/triplo-enhancers, *Su(var)205* and *Su(var)3–7*, indicating that these heterochromatic modifiers also act dose dependently in PDS. Here we use a spontaneous derivative mutation of *Pci* called *Pci^E1^* (*E1*) that variegates like PDS in the absence of *P* elements, presumably due to an adjacent *gypsy* element insertion, to screen for second-site modifier mutations that enhance variable silencing of *white* ^+^ in *E1*. We isolated 7 mutations in *CG8878*, an essential gene, that enhance the *E1* variegated phenotype. *CG8878*, a previously uncharacterized gene, potentially encodes a serine/threonine kinase whose closest *Drosophila* paralogue, *ballchen* (*nhk-1*), phosphorylates histones. These mutant alleles enhance both PDS at *E1* and Position Effect Variegation (PEV) at *w^m4^*, indicating a previously unknown common silencing mechanism between the two.

## Introduction

In *Drosophila melanogaster*, expression of the *white^+^* gene (*w^+^*) is cell autonomous, and necessary for the import of pigment precursors for normal colour in the adult eye. In *white^−^* mutants, the absence of pigment results in a white-eyed phenotype that can be rescued with *w^+^* containing *P*-element transgenes. However, in some insertion locations, expression of *w^+^* is sensitive to the local chromatin environment, such as adjacent heterochromatin. For example, *P{lacW}ciD^plac^* (hereafter *Pci*), a transgene inserted proximally on chromosome 4 between *Ribosomal protein S3A* (*RpS3A*) and *cubitus interruptus (ci)*, was originally isolated as an enhancer trap of *ci* [1]. Its *w^+^* minigene is sensitive to changes in gene dosage for heterochromatin proteins HP1 and SU(VAR)3–7 [2].

The *Pci* transgene is also sensitive to the presence of *P* elements in the genome in a phenomenon called *P* element dependent silencing (PDS), which is phenotypically similar to heterochromatic position effect variegation (hPEV). In flies lacking *P* elements (*M* strains) the *w^+^* transgene is expressed in a uniform manner (even red eye phenotype). However, in flies containing *P* elements (P strains) or *KP* s (derivative elements capable of mimicking some of the characteristics of P strains such as modifying P- repressor sensitive alleles, but not enabling *P* element transposition) variegated expression occurs resulting in a mosaic expression of white ommatidia on a red background in the eye [3]. PDS also occurs when other *w^+^* transgenes are inserted near this location [2].

The random silencing of the *w^+^* minigene in *Pci* during development indicates a phenomenon similar to heterochromatin spreading in hPEV. This is supported by *Pci* responding in a dose sensitive manner to *Su(var) 205* and *3–7*, like *w^m4^* and other centromeric PEV lines [4], [5]. As with PEV, position is important as insertions of the same transgene in other locations do not display PDS. Also, chromosomal translocations of *Pci* away from its centromere-proximal location reduces PDS (Bushey & Locke 2004)2. The position dependence and variegated phenotype suggest PDS occurs via a similar mechanism as heterochromatic PEV.

During investigation of PDS at *Pci* two spontaneous mutants, *P{lacW}ci^DplacE1^* and *P{lacW}ci^DplacE2^* (hereafter *E1* and *E2*), were recovered [2] that showed a variegated eye phenotype in the absence of other *P* elements and a complete white eye phenotype when combined with *P* elements, such as *P{ry*+ *SalI}89D*. PEV modifier loci, such as *Su(var)205* and *Su(var)3–7*, suppressed variegation at *E1* and *E2*. Molecular analysis of *E1* and *E2* revealed each had a novel *gypsy* element insertion approximately 1 kb distal from the *Pci* insert, but in opposite orientations 547 bp apart. Testing of *E1* and *E2* against mutants of *su(Hw)* and *mod(mdg4)* showed that variegation by *E1* and *E2* was not a result of the *gypsy* insulator function *per se* [2]; however, a possible interaction between the *gypsy* insulator and the *wari* element at the 3′ end of the *w^+^* transgene can not be ruled out [6]. Both *E1* and *E2 trans*-silence *w^+^* expression of *Pci* on a paired homolog, but not when present on translocations. Therefore position or pairing contributes to trans-silencing similar to the dominant *trans*-inactivation of the wild-type homologue in *bw* ^+^ */bw^D^* heterozygotes [2], [7], [8], [9].

A previous screen for genetic modifiers of PDS [2] required tracking two chromosomes. In the work described here we have taken advantage of the *E1* variegating PDS system, which is simpler, as all components are on the same chromosome. Furthermore, its intermediate variegated eye phenotype can visibly reveal second site genetic enhancers (white eye phenotype). We describe here the isolation and characterization of seven mutants, that enhance both PDS and hPEV at *w^m4^*. This gene, *CG8878*, was recovered along with mutations in *trithorax* and *ash1 and* appears to encode a novel type of kinase similar to the Vaccinia-Related-Kinase(*VRK*), Casein-Kinase (*CK*), and Tau-Tubulin-Kinase (*TTK*).

## Materials and Methods

### Drosophila stocks and mutations

Unless otherwise cited, *D. melanogaster* mutations were described previously [10]. The *P{lacW}ci^Dplac^ (Pci)* allele (Eaton & Kornberg 1990)1 is a *P{lacZ^P\T.W^w* ^+*mC*^ *amp^R^ ori = lacW}* construct inserted ∼3 kbp upstream (distal) from the *ci* locus on chromosome *4*. *P{lacW}ci ^DplacE1^*(*E1*) has a gypsy element insertion ∼1 kb further upstream as previously described [2]. y^1^ w* P{lacW}3-76a is a *lacW* transgene inserted on the X-chromosome and was originally isolated by Y. N. Jan and provided by the Bloomington stock center, while *In(1)w^m4^,w^m4^* was obtained from K.D. Tartof. Fly stocks were maintained at room temperature on standard yeast/cornmeal medium.

Mutagenesis used *w^−^; dp^−^; e^−^; E1* males treated with 25 mM EMS as per [11] mated to *y^−^ w^−^; +/+* virgin females and screened for a dominant enhanced eye colour phenotype in the progeny. Putative mutants were mated to *w^−^; dp^−^; e^−^; Pci* flies to confirm transmission and segregation and to determine chromosomal location. Mutations were crossed *inter se* to establish recessive lethal complementation groups. Mutant *CG8878* alleles were kept as balanced stocks with *CyO*.

### Genetic Mapping

The dominant enhancer phenotype in an *E1*/*Pci* background was used for genetic recombination mapping because it gave a fuller red eye phenotype, which provided more room for enhancement and thus allowed a more reliable visual assessment of enhancement. Mutants were mapped relative to *wg^Sp^ L Bc* and *Pin* markers. Recombinants both left and right of the enhancer were collected and tested for retention of the enhancer phenotype by crossing males to *w^−^; dp^−^; e^−^; E1* virgin females, and for retention of the recessive lethal phenotype by crossing to other members of the same complementation group. After establishing absolute linkage between the recessive lethal and dominant enhancer phenotypes, the position of the lethal locus was refined by complementation analysis against deficiencies in the region. At least 100 progeny were scored and if the heterozygous mutant/deficiency combination did not occur the combination was considered lethal.

### DNA sequencing

A series of overlapping *CG8878* gene segments were amplified by PCR and the product was sequenced. Point mutations were identified as double peaks on the chromatogram. All polymorphisms and mutations were confirmed by sequencing both strands.

### Eye pigment assays

The amount of *w^+^* gene activity was assayed by measuring the amount of brown eye pigment using a modification of the method of [12]. Heads from 5–9 day old adult flies were stored at −20° until extracted. For each genotype, three replicate samples of 10 heads were extracted in 200 µL of acidified ethyl alcohol (1% HCl in 30% ethanol) with shaking for 48 hours. Absorbance at 470 nm was then measured using a 96 well Costar flat bottom plate in a Bio-Tek PowerWave XS spectrophotometer. Photographs of representative adult flies eyes under mineral oil were taken using a Zeiss stereo microscope and a Nikon Coolpix 995 digital camera. For both the eye pigment assay and the adult eye photographs, the balancer chromosome *CyO* was used as the control.

## Results

### Screen for second site enhancers of *w^+^* variegation in *E1*

We screened ∼44,000 progeny from EMS treated fathers for dominant enhancement of *w^+^* silencing in *E1* and recovered 58 confirmed mutations. *Inter se* recessive lethal complementation analysis showed they fell into five simple and three more complex recessive lethal complementation groups, as well as many singles. Along with mutations in *trithorax* and *ash1* (manuscript in preparation), there was a simple group, with 7 alleles (*1a27a*, *3a22a*, *3a52a*, *3a66a*, *3a90a*, *3a97a*, *4a7a*), which is described here. These seven were examined further and the dominant enhancer of *E1*/*Pci* phenotype for allele *4a7a* genetically mapped to 2–65.4 (n = 490) by recombination relative to *wg^Sp^ L Bc* and *Pin* markers. Linkage between the dominant enhancer of *E1*/*Pci* and recessive lethal phenotypes was demonstrated as described in the Materials and Methods. Deficiency mapping of the lethal phenotype of this group refined its position to 48E2;48E4, within *Df(2R)BSC199* (7,779,605: 8,059,989) but not *Df(2R)BSC879* (7,779,605: 8,029,867), which includes the predicted *CG8878* gene. This is the only candidate gene in this region predicted to influence gene expression in a heritable manner, expressed in the correct tissue at the correct time, and of a size likely to result in 7 independent mutations in a mutagenesis of this size. We note that both *Hen1* and *Prp8* might influence gene expression post-translationally, which should not lead to silencing in a clonal manner (variegation) as seen here.

### DNA sequencing of the mutants

**Figure 1:**
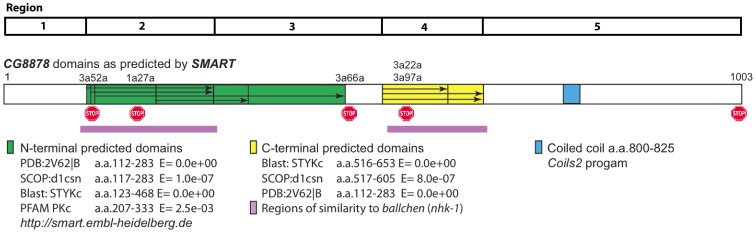
Schematic representation of *CG8878* polypeptide sequence showing domains predicted by SMART (University of Heidelberg) and the location of lesions described in this study. Mutant designations are above the polypeptide backbone while the nature of the corresponding mutation is below. Regions of sequence similarity to *ballchen* (*nhk-1*) are shown as mauve bars below the CG8878 sequence. The five regions identified in the dot plots are shown above the polypeptide diagram.

DNA sequencing spanning the entire predicted coding region of *CG8878*, in heterozygotes with the *CyO* balancer chromosome, showed that five alleles (*1a27a*, *3a22a*, *3a52a*, *3a66a*, *3a97a*) had a base pair change within *CG8878* that altered the predicted amino acid coding sequence (Table 1, Figure 1). Three of the alleles (*3a27a*, *3a52a* and *3a97a*) had G/C to A/T transitions that resulted in premature stop codons; with *3a52a* being at the amino terminal end of the first predicted STKc domain and therefore likely to be a null allele. Allele *3a66a* had a single nucleotide deletion that caused a frame-shift leading to multiple premature stop codons while *1a27a* had a G/C to A/T transition that predicts the loss of an intron donor splice site, a frame-shift and multiple premature stop codons. Two other alleles (*3a90a* and *4a7a*) had identical nineteen base pair deletions in the 5′ upstream promoter region that included 4 base pairs of the proximal predicted E box and are thus presumptive transcriptional regulatory mutants.

**Table 1:** List of EMS induced mutations in CG8878, their DNA sequence changes, and their predicted changes to the amino acid sequence.

Mutant	Mutation	Type	Predicted amino acid change
1a27a	G→A 8037095	Loss of intron donor splice site, frameshift.	Insert 130–180Opal
*3a22a*	C→T m1942	Point, transition.	R546Opal
*3a52a*	G→A m675	Point, transition.	W123Opal
*3a66a*	1Δ C m1665	Frameshift.	454–468Amber
*3a90a*	19 bp Δ T 8,038,801-19	5′ regulatory deletion.	4 bp Δ E box
*3a97a*	C→ T m1942	Point, transition.	R546Opal
*4a7a*	19 bp Δ T 8,038,801-19	5′ regulatory deletion.	4 bp Δ E box

Table showing mutagen used, coding sequence change, type of mutation, and resulting effective amino acid alteration of the mutant.

### Phenotypic characterization of the mutants

**Figure 2:**
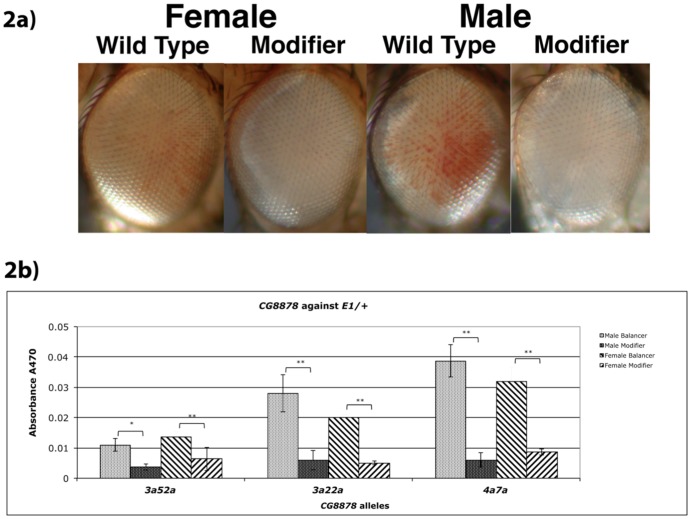
Enhancement of *E1* by CG8878 mutations. a) Representative photographs of eyes from each class of progeny from heterozygous *CG8878* mutants crossed back to the parental *E1* stock used in this mutagenesis. *Cy* versus *Cy^+^* flies were compared for each sex. Flies are posed facing right. (*y^−^ w^−^*; *dp^−^ 3a52a*/*CyO, Cy dp^−^* ♂ X *w^−^*; *dp^−^*; *e^−^*; *E1* 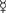). b) Pigment assays of heterozygous *CG8878* mutants crossed back to the parental *E1* stock used in this mutagenesis. *Cy* versus *Cy^+^* flies were compared for each sex. (*y^−^ w^−^*; *dp^−^ CG8878*/*CyO, Cy dp^−^*♂ X *w^−^*; *dp^−^*; *e^−^*; *E1* 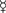) Statistical significance between pairs (T-Test = 1-tailed, independent (unpaired, unequal variance)) is given above each mutant pair tested, _*_ = p<0.05, _**_ = p<0.01, NS = not significant.

Visual pigment assessment for the dominant enhancement of white-eyed variegation in *E1/+* heterozygotes indicated all mutant alleles were enhanced relative to the *CyO* control in both sexes, and frequently produced flies indistinguishable from *w^−^*. Representative photographs of mutant eyes are given in Figure 2a. The extent of enhancement was quantified by pigment assays. Both male and female mutants had less than half the pigment of non-mutant internal control flies (*CyO* balancer) from the same cross, with all being significant (95% confidence limit - Fig. 2b). All mutant alleles enhance silencing of *E1/+*.

**Figure 3:**
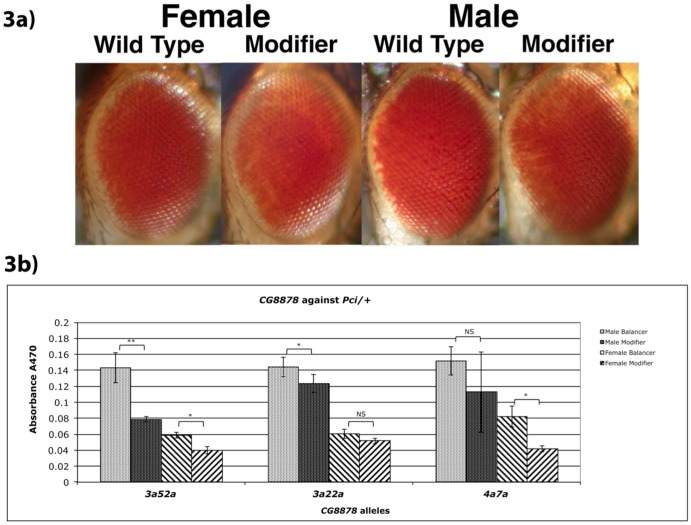
Enhancement of *Pci* by *CG8878* mutations. a) Photographs of representative examples from each class of progeny from heterozygous *CG8878* mutants crossed back to *Pci*, the parental stock from which *E1* was derived. *Cy* versus *Cy^+^* flies were compared for each sex. Flies are posed facing right. (*y^−^ w^−^*; *dp^−^ 3a52a*/*CyO, Cy dp^−^* ♂ X *w^−^*; *dp^−^*; *e^−^*; *Pci* 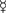). b) Pigment assays of heterozygous *CG8878* mutants crossed back to the parental *Pci* stock *E1* was derived from. *Cy* versus *Cy^+^* flies were compared for each sex. (*y^−^ w^−^*; *dp^−^ CG8878*/*CyO, Cy dp^−^* ♂ X *w^−^*; *dp^−^*; *e^−^*; *Pci* 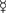) Statistical significance between pairs (T-Test = 1-tailed, independent (unpaired, unequal variance)) is given above each mutant pair tested, _*_ = p<0.05, _**_ = p<0.01, NS = not significant.

To see if the dominant enhancement (*w^+^* silencing) was limited to the *E1* allele, we quantitatively assayed the effect of *the* mutants on *Pci*/+ flies, which lack the gypsy element present in *E1*. All mutants reduced the amount of pigment compared to non-mutant internal control flies (*CyO* balancer) from the same cross (Figure 3b). However, only the putative null allele *3a52a* showed significance in both sexes. Visually, variegation was less visible in this cross, with eyes displaying only a pattern of weak silencing starting at the posterior edge, with rapidly decaying anterior progression (Figure 3a).

**Figure 4:**
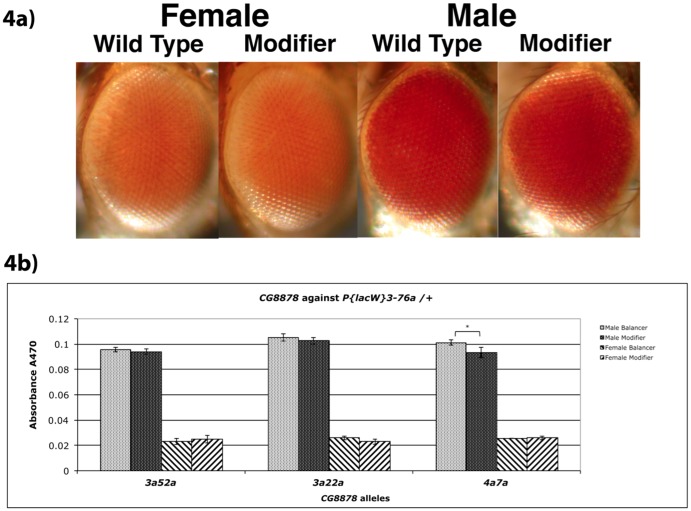
Enhancement of *P{lacW}3-76a* by CG8878 mutations. a) Photographs of representative examples from each class of progeny from heterozygous *CG8878* mutants crossed to *P{lacW}3-76a*, an insertion of the same transgene as *Pci*, but only at 18A1 on the X chromosome. *Cy* versus *Cy^+^* flies were compared for each sex. Flies are posed facing right. (*y^−^ w^−^*; *dp^−^ 3a52a*/*CyO Cy dp^−^* ♂ X *y^1^ w^−^ P{lacW}3-76a* 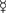). b) Pigment assays of heterozygous *CG8878* mutants crossed to *P{lacW}3-76a*. *Cy* versus *Cy^+^* flies were compared for each sex. (*y^−^ w^−^*; *dp^−^ CG8878*/*CyO Cy dp^−^* ♂ X *y^1^ w^−^ P{lacW}3-76a* 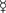) Statistical deviation between pairs was insignificant (T-Test = 1-tailed, independent (unpaired, unequal variance)) except for *4a7a* males which was significant at p<0.05 (_*_ = p<0.05).

To address the possibility that these mutations were acting directly on the w+ transgene, and not via any dependence on insertion position, we assayed pigment in y^1^ w* P{lacW}3-76a/+ flies with these mutants. The transgene in *P{lacW}3-76a* is the same as that in *Pci* but is located at 18A1 (60.7 cM) on the X chromosome and presents a full red eye. Of the three alleles tested, only 4a7a, a putative regulatory mutant, males showed a barely significant difference (p = .03) from non-mutant control flies (*CyO* balancer) of the same cross indicating that the dominant enhancement (silencing) is dependent upon chromosomal location and not on the *w^+^* transgene *Pci* construct itself (Figure 4). The consistency of our results across all mutants tested indicates that this method of pigment determination is both accurate and precise.

**Figure 5:**
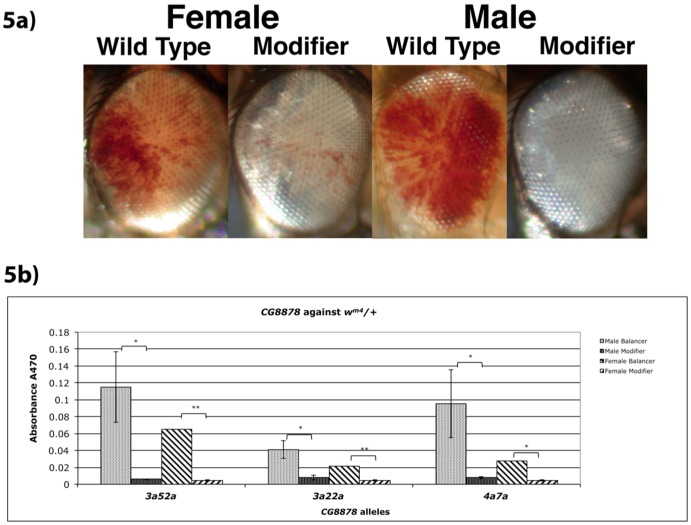
Enhancement of *w^m4^* by CG8878 mutations. a) Photographs of representative examples from each class of progeny from heterozygous *CG8878* mutants crossed to *w^m4^*. *Cy* versus *Cy^+^* flies were compared for each sex. Flies are posed facing right. (*y^−^ w^−^*; *dp^−^ 3a52a*/*CyO*, *Cy dp^−^* ♂ X *w^m4^*; *dp^−^*; *e^−^* 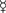). b) Pigment assays of heterozygous *CG8878* mutants crossed to *w^m4^*. *Cy* versus *Cy^+^* flies were compared for each sex. (*y^−^ w^−^*; *dp^−^ CG8878*/*CyO Cy dp^−^* ♂ X *w^m4^*; *dp^−^*; *e^−^* 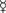) Statistical significance between pairs (T-Test = 1-tailed, independent (unpaired, unequal variance)) is given above each mutant pair tested, _*_ = p<0.05, _**_ = p<0.01, NS = not significant.

Next, we asked whether these mutants had an effect on classical hPEV by crossing *y− w−*; dp− CG8878*/CyO, Cy dp− males to virgin *In(1)w^m4^*; *dp−; e−* females. Variegation of *w^m4^* was visibly enhanced by all three mutants tested (Figure 5 a) and quantitatively (95% and 99% confidence limits) enhanced in male and female flies respectively (Figure 5b).

### Amino acid sequence comparisons

**Figure 6:**
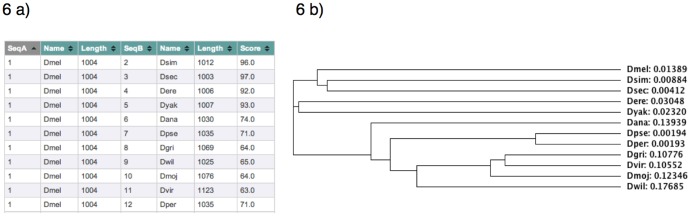
Comparison between *D. melanogaster* CG8878 amino acid sequences and those of eleven other Drosophila species. Accession numbers given in Table S1 and the alignment in Figure S1. a) Score table showing degree of similarity between CG8878 homologues. b) Cladogram showing relative evolutionary distances between CG8878 homologues for twelve Drosophila species (http://www.ebi.ac.uk/Tools/msa/clustalw2/). Names are abbreviated using the capitalized first letter of the genus followed by the first three letters of the species.

Analysis of *CG8878'*s predicted polypeptide sequence using SMART (University of Heidelberg) predicts two domains related to protein kinase separated by 194 amino acids (Figure 1). A comparison of *CG8878*'s predicted amino acid sequence with eleven other Drosophila species reveals that homologs are present and highly conserved in all twelve species studied; this supports *CG8878* being an essential gene (Figure 6, Figure S1). This cladogram parallels that already determined for these species [13].

**Figure 7:**
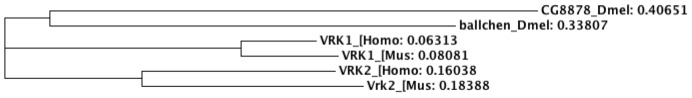
Phylogram showing evolutionary distances between *CG8878*, *ballchen* and mammalian *VRK1 and VRK2*. Abbreviations are as follows: *Drosophila melanogaster* (Dmel), *Homo sapiens* (Homo), *Mus musculus* (Mus) (http://www.ebi.ac.uk/Tools/msa/clustalw2/) Accession numbers given in Table S1.

**Figure 8:**
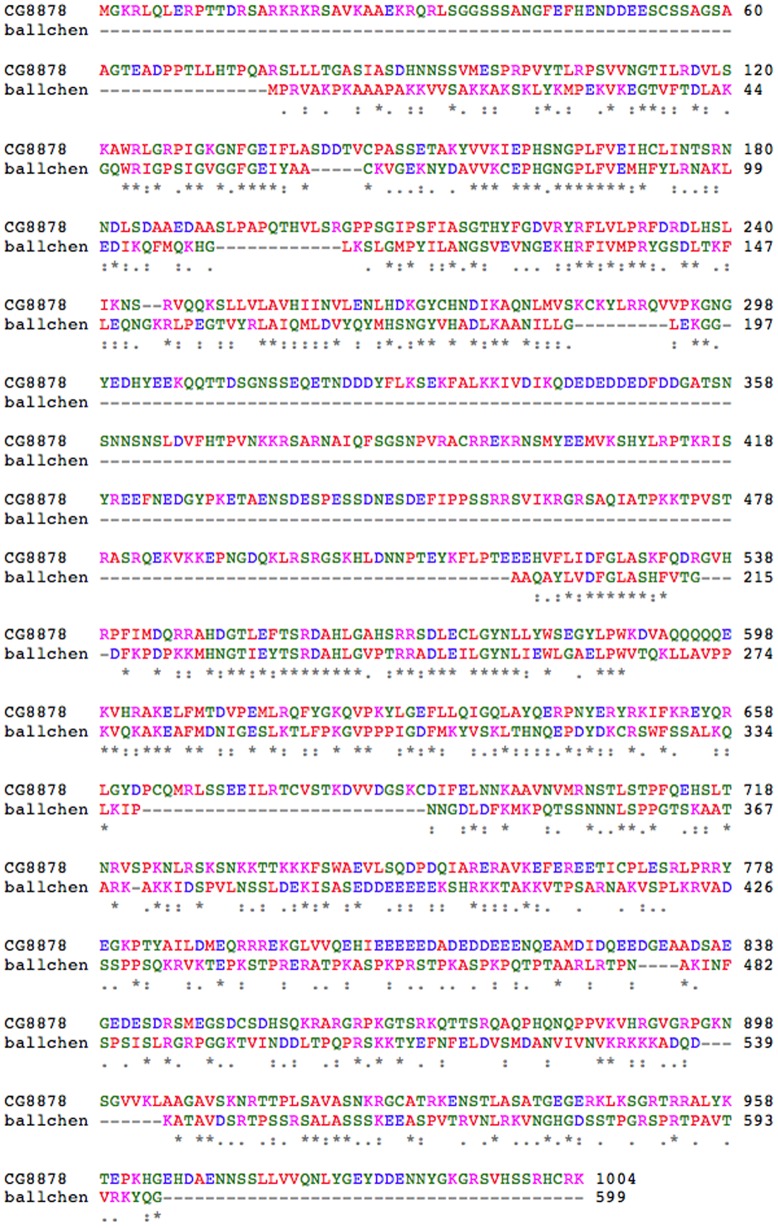
Pairwise alignment of CG8878, and BALLCHEN, its closest *Drosophila* paralogue. Comparison symbols: * = identity, : = side groups with strongly similar properties, . = side groups with weakly similar properties. Amino acid color code: red = small hydrophobic, blue = acidic, magenta = basic, green = hydroxyl, sulfhydryl, amine, G. • Accession numbers given in Table S1.

The amino acid sequence of *CG8878* shows the most similarity to *D. melanogaster ballchen*, and human orthologs, Vaccinia Related Kinases (VRK1, VRK2), which encode a nucleosomal histone 2a kinase (Figure 7, Figure S2). Amino acid sequence comparisons suggest both *CG8878* and *ballchen* are derived from a common *VRK* like precursor. However, in *CG8878*, the VRK domain appears to have been split in two by an ∼194 amino acid insertion (Figures 1, 8). CG8878 shows 36% identity and 56% positive correlation to BALLCHEN over both parts of the kinase domain (http://blast.ncbi.nlm.nih.gov/Blast.cgi) indicating a functional conservation. Note, since our mutation *3a22a* (R546Opal) is recessive lethal and enhances variegation at *E1*, *Pci*, and *w^m4^* (Figures 2 b, 3 b, 5 b), it appears the second part of *CG8878*'s split VRK-like kinase domain (Fig. 8) is essential for *CG8878*'s function.

### Structure of *CG8878*

**Figure 9:**
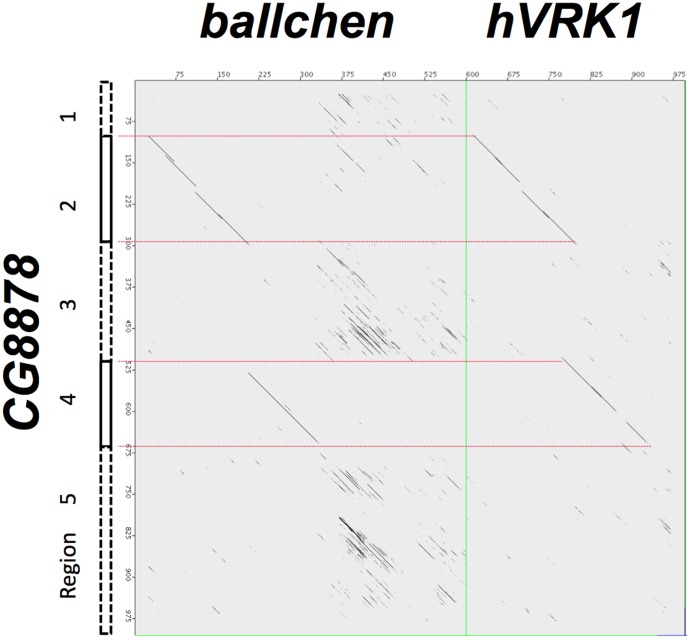
JDotter dotmatrix comparison [25] between the amino acid sequences of *D. melanogaster CG8878* (NP_610733.1) and *ballchen* (NP_651508.1) and human VRK1 (NP_003375.1). The single PcK domain in *ballchen* and *hVRK1* are split in *CG8878*. Horizontal, red dotted lines mark the five regions, with 2 and 4 corresponding to the conserved PcK functional domain. The vertical, green line separates ballchen and hVRL1 sequences. Setting for JDotter are: Window size: 50; Zoom factor: 1 base/pixel; Pixel factor: 48; Scoring matrix: BLOSUM62; GreyMap Tool: 0, 13; Maximum Plot size: 700 bases/pixel; Sliding window size for new plots: 50.

An amino acid sequence similarity is also found with human Casein Kinases and human Tau-Tubulin Kinases. This corresponds to a PcK kinase conserved domain (called PHA02882). However, a dot plot comparison of *CG8878* with *ballchen* and *hVRK1* (Figure 9) shows that the single, conserved PcK domain is split into two regions in *CG8878*. Thus the whole *CG8878* sequence can be separated into 5 regions (1–96, 97–291, 292–516, 517–662, and 663–1004), with 2 and 4 corresponding to the PcK conserved domain.

**Figure 10:**
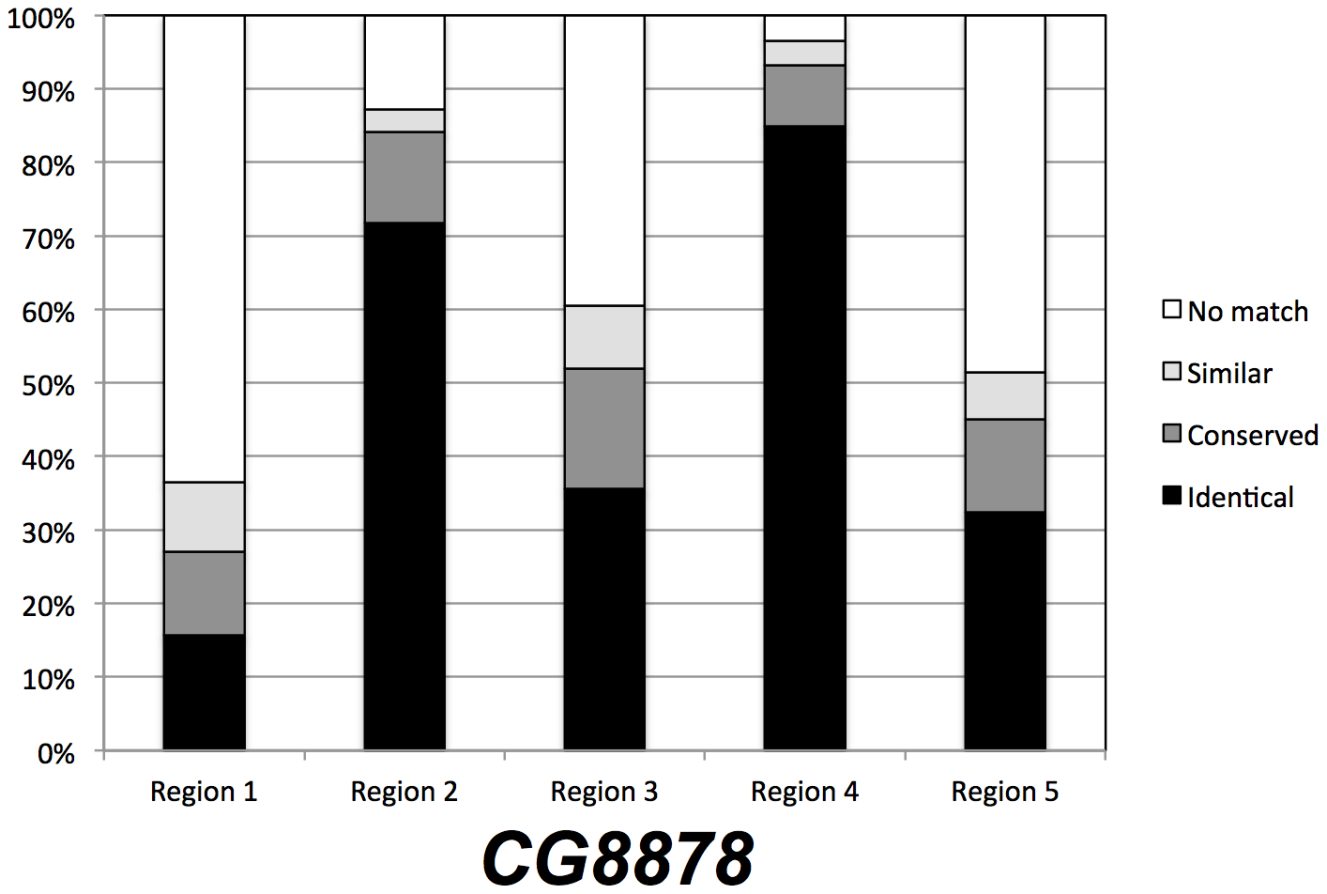
Amino acid sequence similarity in the five regions of *CG8878* among Drosophila species. The twelve orthologs of the D. melanogaster *CG8878* (see Figure S1) were aligned using ClustalW and the sequence similarities were counted as identical, conserved, similar, or no match for each of the five regions and is shown as a percent of the total in that region. Regions 2 and 4, which correspond to the PcK domain conserved with *ballchen* and *hVRK1*, are more conserved among these orthologs than the other regions.

A ClustalW sequence alignment of *D. melanogaster CG8878* with eleven other Drosophila orthologs all show a similar split PcK domain and 5 region organization (Figure S1). Comparison of sequence variation in each of the 5 regions shows that regions 2 and 4 are more conserved among these orthologs, suggesting evolutionary sequence conservation (Figure 10).

**Figure 11:**
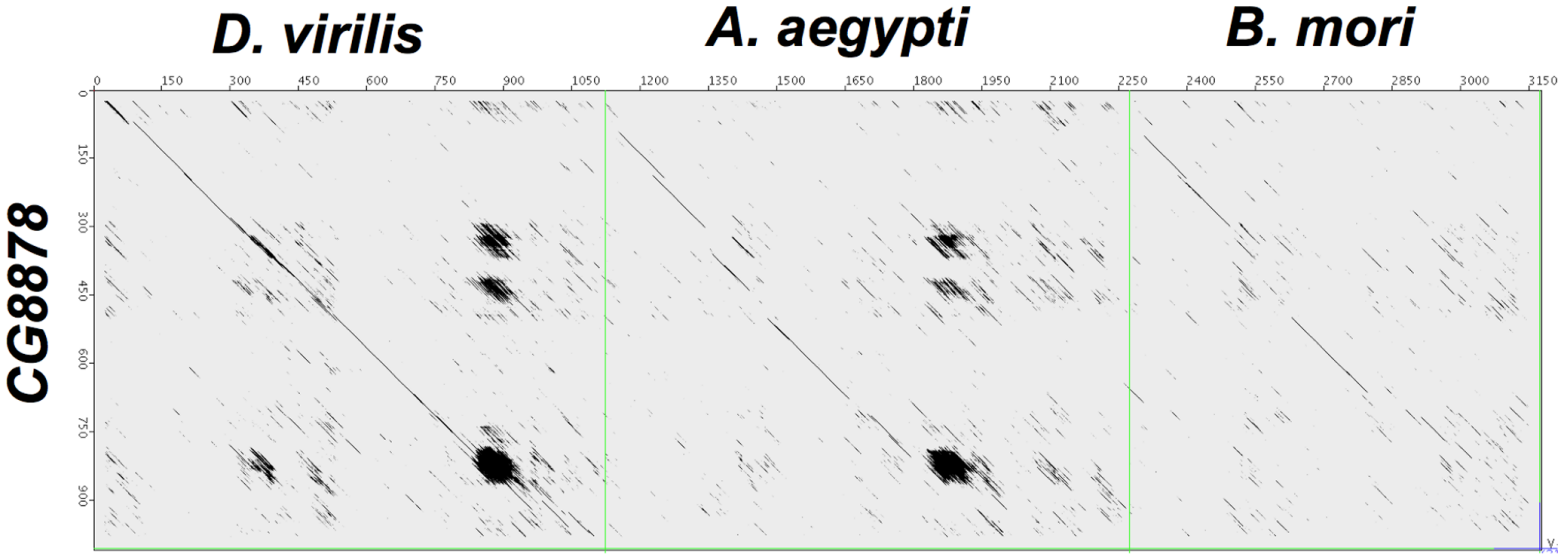
JDotter dotmatrix comparison [25] between the amino acid sequences of *D. melanogaster CG8878* (NP_610733.1) and orthologs from *D. virilis* (XP_002050972.1), *A. aegypti* (EAT48618.1), and *B. mori* (BGIBMGA000118-PA_www.silkdb.org). The three repeated, aspartic and glutamic acid rich regions are present in all Diptera but absent from *B. mori*, a moth. Setting for JDotter are as in Figure 9, but with Maximum Plot size: 1200 bases/pixel.

Dot plot analysis, comparing *D. melanogaster* with *D. virilis* (Figure 11), also shows three regions of repeated sequence that are rich in glutamic and aspartic acid. Repeat 1 (position 300–353) has 42%, repeat 2 (421–449) has 46%, and repeat 3 (790–854) has 50% glutamic and aspartic acid content. Similarly positioned repeats can be found in three mosquito genes (*A. aegypti*, *A. gambiae*, and *C. quinquefasciatus*) supporting the contention that they are orthologs. *Bombyx mori*, a moth, has a similar gene (BGIBMGA000118-PA_www.silkdb.org) with a split PcK domain, but it lacks the three acid-rich regions. No other split PcK domain genes were identified; all other PcK containing genes had single, uninterrupted domains.

**Figure 12:**
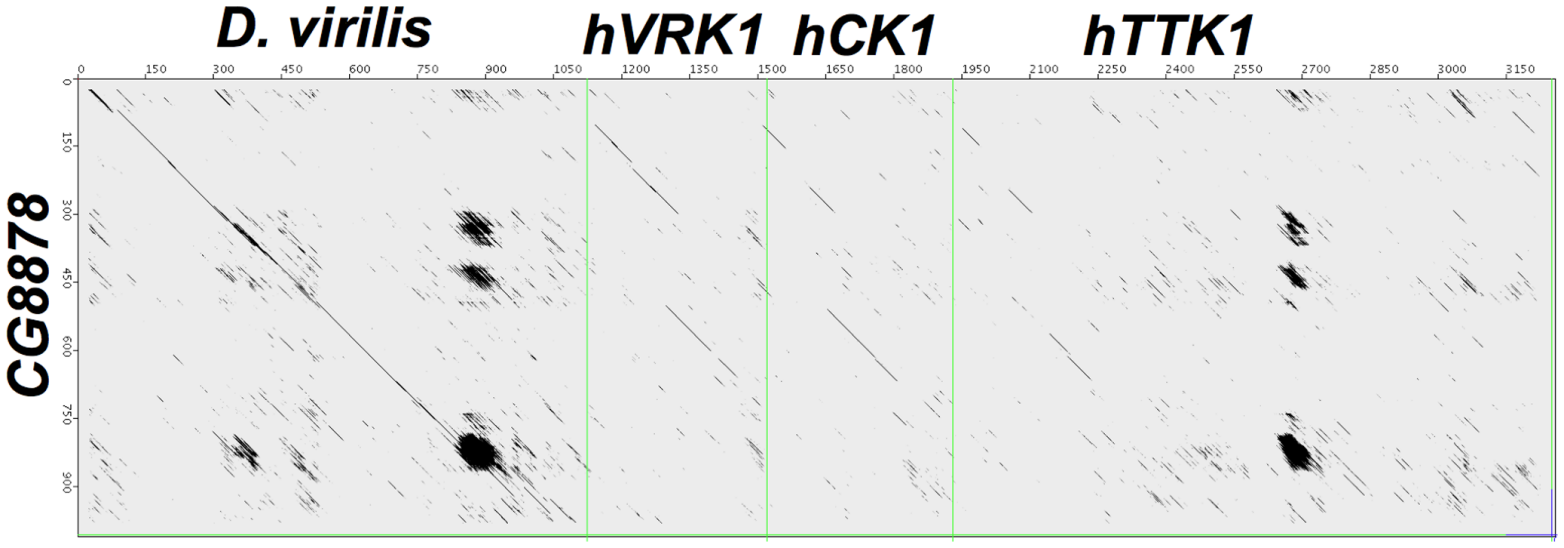
JDotter dotmatrix comparison [25] between the amino acid sequences of *D. melanogaster CG8878* (NP_610733.1) and orthologs from *D. virilis* (XP_002050972.1), human *VRK1* (NP_003375.1), human CK1 (CaseinKinase 1; NP_620693.1), and human TTK1 (Tau-Tubulin Kinase 1; NP_115927.1). The three repeated, aspartic and glutamic acid rich regions are present in hTTK1 but not hVRK1 or hCK1. Setting for JDotter are as in Figure 11.

Additional dot plot analysis of *CG8878* with *hVRK1*, *hCK1*, and *hTTK1* (Figure 12) shows that *hVRK1* and *hCK1* have single PcK domains and lack a long C-terminal region. *hTTK1* also has a single PcK domain, but contains an acid-rich repeat region, like *CG8878*. The *D. melanogaster* ortholog of *hTTK1* is *asator* [14], which has a single PcK domain in the N-terminal quarter of the polypeptide, and lacks any acid-rich repeat regions in the C-terminal three-quarters end.

**Figure 13:**
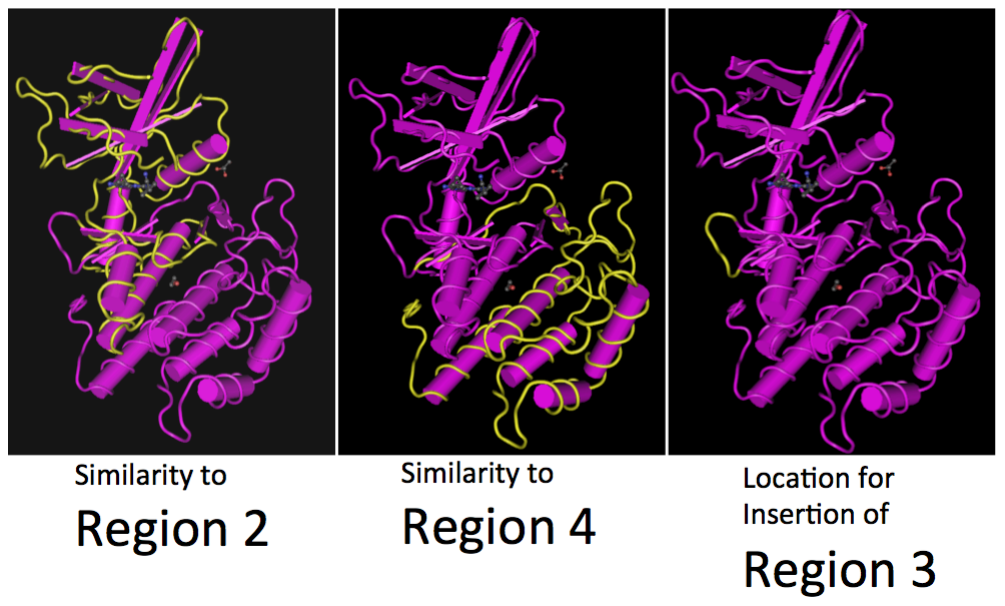
Three dimensional structure diagrams, as determined by Nuclear Magnetic Resonance, of human hVRK1 (file 3OP5) displayed using Cn3D4.3 software [26]. Regions of hVRK1 that are similar to *D. melanogaster CG8878* are highlighted in yellow. Regions 2 (left) and 4 (centre) correspond to the conserved sequence regions. The location between these two regions, corresponding to the insertion of Region 3 (right) is on the surface of the polypeptide and highlighted in yellow.

By examining the 3D hVRK1 structure as determined by Nuclear Magnetic Resonance [15] and the linear alignment of *CG8878* with *hVRK1* sequences, the splitting of the PcK domain in *CG8878* would correspond to an insertion of Region 3 after part of the catalytic loop (just beyond the putative Ser/Thr kinase active site) but before the activation loop. This location is at the surface of the structure and corresponds to position 187–191 (YKNPD) in *hVRK1* (Figure 13).

NucPred [16] predicts, with a score of 1.0, a nuclear localization signal (NLS) in *CG8878* at position 17–21 (RKRKR). This sequence is present in a similar position in all 12 Drosophila orthologs (Figure S1), suggesting it is conserved. Similar NLS sequences are present, but at different locations, in the three potential mosquito orthologs (score 0.95–1.0). Discrete NLS sequences appear absent from the *B. mori* gene (Score 0.87).

We conclude the hybrid nature (kinase sequence most similar to *ballchen*/*hVRK1* and presence of three acid rich repeats like that in *hTTK1*) and split PcK domain of *CG8878* defines a novel kinase type to be added to the VRK, CK, and TTK groups.

## Discussion

We induced, recovered, and characterized seven mutations that dominantly enhance the variable silencing (variegation) of *E1*, whose expression is similar to *P* element dependent silencing (PDS). The dominant enhancement genetically maps at or near the *CG8878* locus and it could not be separated from the lethal phenotype by crossing over. The lethal phenotype deficiency maps to a very fine region that includes *CG8878*. Five alleles contain mutations resulting in stop codons; two at the amino terminal end of CG8878's amino proximal predicted STKc domain likely represent null alleles, one between CG8878's two predicted kinase domains, and two in the amino end of *CG8878*'s carboxy proximal predicted kinase domain. Taken together, this shows that loss of the *CG8878* gene function is responsible for the dominant enhanced silencing of *w^+^* in *E1* and a recessive lethal phenotype. Bioinformatic analysis of *CG8878* indicates that it is likely a protein kinase, but the putative functional domain has been split in two. Furthermore, this split form appears limited to Dipterans.

### *CG8878* and *Hen1*

The *CG8878* transcription unit is located entirely within the large (5.4 kb) second intron of another gene, *Hen1* (formerly *Pimet*), in the antisense orientation. *Hen1* has been shown to mediate 2′-*O*-methylation at the 3′ end of *Piwi* interacting RNAs in *Drosophila* [17], [18]. *Piwi* interacting RNAs are germ-line specific 24–30 nt RNAs that couple with PIWI proteins to silence invading transposable elements (reviewed by [19]). Given that *Pci* has *P* element terminal repeats and, at the 5′ end, a *P* element transposase *lacZ* fusion, we considered that *Hen1*, and not *CG8878*, might potentially be the enhancer identified in this screen, but several points argue against this: 1) all seven mutants had lesions in *CG8878* coding or regulatory sequences; 2) all of these lesions are entirely inside *Hen1*'s second intron, and predict no effect on *Hen1* expression; 3) Hen1 is not an essential gene because *PBac(WH)Hen1[f00810]* is a null for *Hen1* [18] but is not recessive lethal; 4) *P{lacW}3-76a* appears to be unaffected by our *En*(*var*)s despite being the same construct only at a different location; and 5) *w^m4^*, which is not *P* element derived, is significantly affected by our *En*(*var*)s. The most parsimonious explanation is that these mutations are due to lesions in *CG8878*, not *Hen1*, and that *CG8878* is an essential gene and when mutated has a dominant *En*(*var*) phenotype.

### Potential molecular function of *CG8878*

Although we have been unable to find split kinase domain *CG8878* homologues outside of the order Diptera, *CG8878* is highly conserved across Drosophila species (Figures 6, Figure S1). Nevertheless, the conservation of both VRK like kinase domains indicates that it is likely to encode a kinase of unknown, but essential, specificity. The closest *Drosophila melanogaster* paralog of *CG8878* is *ballchen* (an NHK-1 homolog), with regions of maximum similarity coinciding with *CG8878*'s putative kinase domains as shown in Figure 1b). *ballchen* has high affinity for chromatin and has been shown to phosphorylate Threonine 119 at the carboxy terminus of nucleosomal, but not free, H2A in *Drosophila* embryos. H2A T119 is phosphorylated during mitosis but not in S phase which coincides with NHK-1's chromatin association as shown by immunostaining and may be a component of the histone code related to cell cycle progression [20]. Ivanovska *et al.* [21] described a point mutation, Z3-0437, in the kinase domain of NHK-1 that led to female sterility due to defects in the formation of the karyosome. This led to metaphase I arrest as a result of failure of the synaptonemal complex to disassemble and to load condensin onto chromosomes in the mutant. Mitosis was also shown to be affected, as embryos laid by *nhk-1* ^−/−^ mutant females arrested with aberrant mitotic spindles and polar bodies. They also found a lack of Histone H4K5 and H3K14 acetylation in the karyosomes in *nhk-1* mutant but not control oocytes, implying that Histone H2A threonine 119 phosphorylation is required for meiotic acetylation of these residues. Lancaster *et al.* [22] found that phosphorylation of *barrier to autointegration factor* protein (BAF) by NHK-1 was necessary for karyosome formation. Loss of NHK-1 or expression of nonphosphorylatable BAF resulted in ectopic chromosome-nuclear envelope association in oocytes leading the authors to propose that tethering of chromosomes to the nuclear envelope is disrupted by NHK-1 mediated BAF phosphorylation, allowing karyosome formation in oocytes.

*CG8878*'s exact target and mode of action are yet to be determined, but sequence similarities suggest that Histone phosphorylation by *CG8878* would readily explain its action as an *En*(*var*). For example, JIL1 phosphorylation of H3S10 blocks methylation of H3K9 allowing hyperacetylation of Histone 3 and promoting a transcriptionally active chromatin state [23]. *CG8878*'s expression profile is consistent with it being a genome wide inhibitor of heterochromatin spread as it is expressed in all tissues, at all stages of development, with maxima at times of peak developmental change, such as early embryogenesis and prepupariation [24].

Our mutants suggest the predicted kinase domains are essential for function. The enhancer (of *E1* and *w^m4^*) phenotypes and recessive lethal phenotypes of *3a66a*, which results in a premature stop codon between *CG8878*'s two predicted kinase domains, and *3a22a*, and *3a97a*, which result in a premature stop codon in the amino end of *CG8878*'s carboxy proximal predicted kinase domain, all argue that this latter predicted kinase domain is essential for *CG8878* function.

The putative Kinase coding region of *CG8878* is most similar to *hVRK1*, but is split into two segments (Regions 2 and 4). The conserved NLS sequence supports nuclear localization and thus a possible role in chromatin modification. The conserved presence of the aspartic and glutamic acid rich repeats suggest possible interaction sites. These are lacking in *hCK1*, a cytosolic protein, only present once in *hTTK1*, and absent in the *D. melanogaster asator* (TTK1 ortholog). Together, this suggests that *CG8878* encodes a protein Kinase that modifies chromatin structure.

### *CG8878* acts at the *ci* locus

*Pci* was isolated as an enhancer trap at the *ci* locus since the enhancer-trap reporter accurately mimicked that of *ci* RNA with both being expressed specifically in anterior compartment cells of the imaginal discs [1]. The *w^+^* transgene in *Pci* (and the *E1* gypsy element) are inserted in the *ci* distal regulatory region. *Pci* is a recessive allele of *ci* because it exhibits *ci* wing phenotype when heterozygous with *ci^57g^* (a deletion upstream of *Pci* in the regulatory region) and *ci^1^* (a gypsy insert upstream of *Pci*). All our mutant *CG8878* alleles enhance variegation (reduce *w*+ expression in the transgene) in *E1* and *E1/Pci* (unpublished observation), but have little effect on *P{lacW}3-76a*, the same construct at a different location. Thus the silencing is location dependent and is thus not likely due to a direct interaction with the *white* promoter, but with the *ci* regulatory region itself. Since *Pci* reporter expression is approximately halved when *3a52a* is present, and does not depend on the presence of *E1*, we infer that *CG8878* normally acts at the *ci* regulatory region to impede the spread of heterochromatin into this region, likely in a dose sensitive manner.

## Supporting Information

Figure S1 **Pairwise alignment of CG8878 and 12** ***Drosophila*** **homologues.** Species names are abbreviated using the capitalized first letter of the genus followed by the first three letters of the species. Comparison symbols: * = identity, : = side groups with strongly similar properties, . = side groups with weakly similar properties. Amino acid color code: red = small hydrophobic, blue = acidic, magenta = basic, green = hydroxyl, sulfhydryl, amine, G (http://www.ebi.ac.uk/Tools/msa/clustalw2/. Note: for *D. persimilis* a nucleotide was removed (five A's to four A's – a presumed sequencing error) to facilitate amino acid alignment. Accession numbers given in Table S1.
(TIF)

Figure S2 **Pairwise alignment of BALLCHEN and VRK1 from mouse and humans.** Symbols are the same as Figure S1. Comparison symbols: * = identity, : = side groups with strongly similar properties, . = side groups with weakly similar properties. Amino acid color code: red = small hydrophobic, blue = acidic, magenta = basic, green = hydroxyl, sulfhydryl, amine, G (http://www.ebi.ac.uk/Tools/msa/clustalw2/.) Accession numbers given in Table S1.
(TIF)

Table S1 **Polypeptide accession numbers used.** Note: the *persimilis* sequence was modified by the removal of one nucleotide from the DNA sequence to alter the amino acid reading frame to facilitate alignment.
(PDF)
