# Supplementary figures and images for: Mutations in CG8878, a Novel Putative Protein Kinase, Enhance P Element Dependent Silencing (PDS) and Position Effect Variegation (PEV) in Drosophila melanogaster
Source: PLoS One. 2014 Mar 10;9(3):e71695. doi: 10.1371/journal.pone.0071695 (PMC3948951; doi:10.1371/journal.pone.0071695)

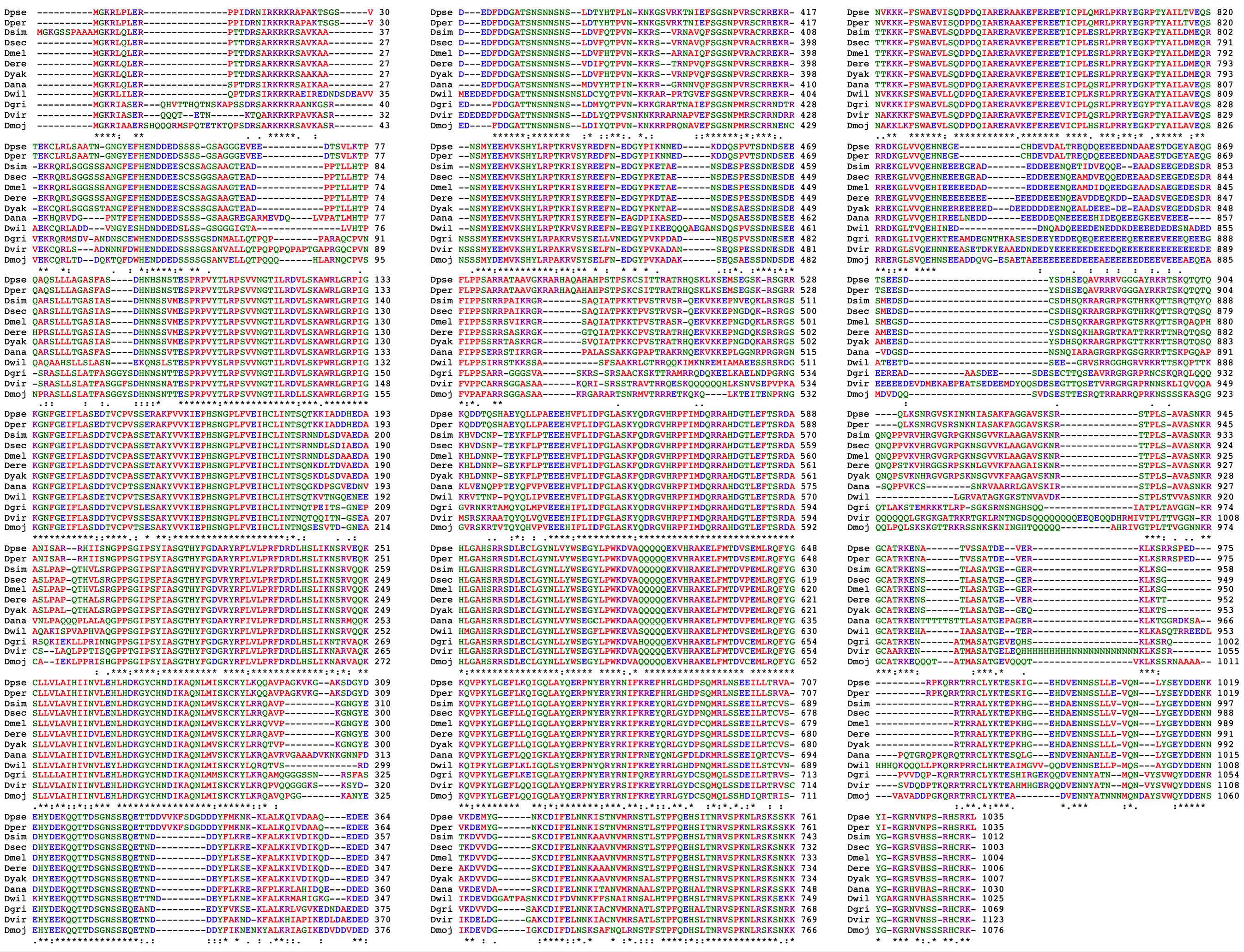

Supplement: Figure S1 — Pairwise alignment of CG8878 and 12 Drosophila homologues. Species names are abbreviated using the capitalized first letter of the genus followed by the first three letters of the species. Comparison symbols: * = identity, : = side groups with strongly similar properties, . = side groups with weakly similar properties. Amino acid color code: red = small hydrophobic, blue = acidic, magenta = basic, green = hydroxyl, sulfhydryl, amine, G (http://www.ebi.ac.uk/Tools/msa/clustalw2/. Note: for D. persimilis a nucleotide was removed (five A's to four A's – a presumed sequencing error) to facilitate amino acid alignment. Accession numbers given in Table S1. (TIF) [file pone.0071695.s001.tif]

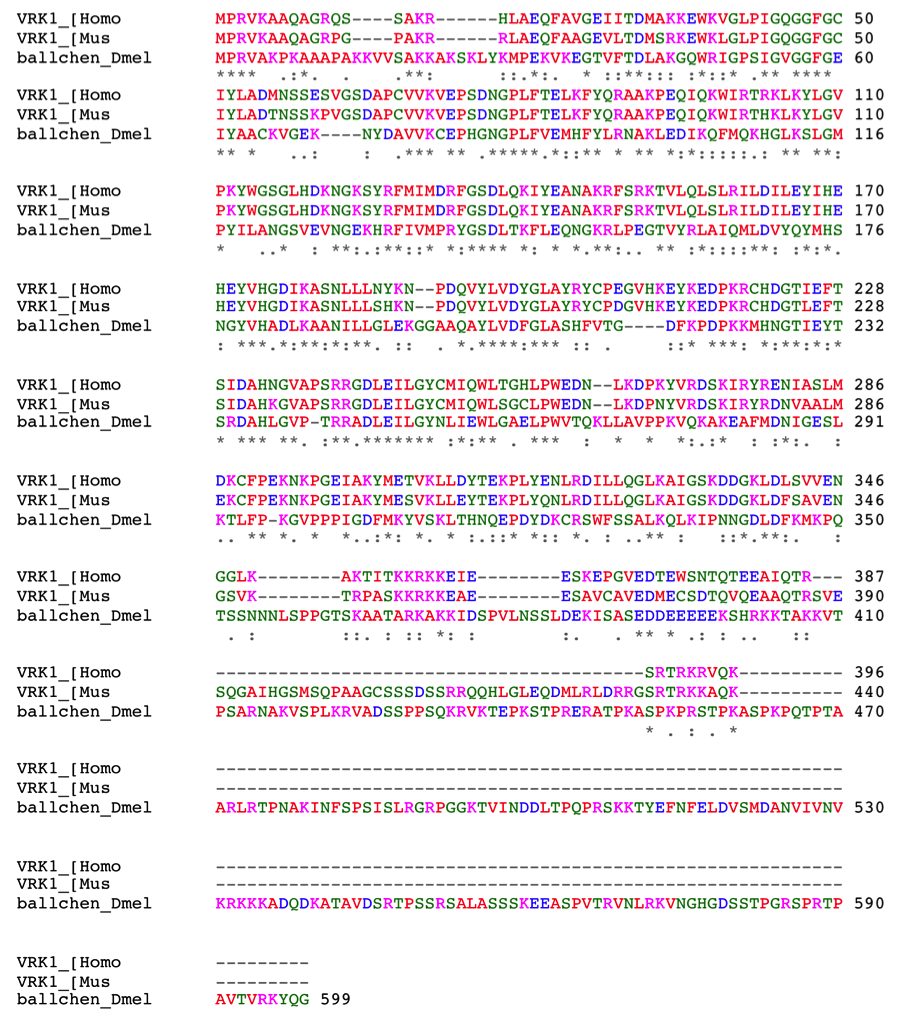

Supplement: Figure S2 — Pairwise alignment of BALLCHEN and VRK1 from mouse and humans. Symbols are the same as Figure S1. Comparison symbols: * = identity, : = side groups with strongly similar properties, . = side groups with weakly similar properties. Amino acid color code: red = small hydrophobic, blue = acidic, magenta = basic, green = hydroxyl, sulfhydryl, amine, G (http://www.ebi.ac.uk/Tools/msa/clustalw2/.) Accession numbers given in Table S1. (TIF) [file pone.0071695.s002.tif]
