# Supplementary material for: Mutations in CG8878, a Novel Putative Protein Kinase, Enhance P Element Dependent Silencing (PDS) and Position Effect Variegation (PEV) in Drosophila melanogaster
Source: PLoS One. 2014 Mar 10;9(3):e71695. doi: 10.1371/journal.pone.0071695 (PMC3948951; doi:10.1371/journal.pone.0071695)
Supplement: Table S1 — Polypeptide accession numbers used. Note: the persimilis sequence was modified by the removal of one nucleotide from the DNA sequence to alter the amino acid reading frame to facilitate alignment. (PDF) [file pone.0071695.s003.pdf]

Supplemental Information:

Table S1

Species	Gene (Protein)	Accession #
Drosophila melanogaster	*CG8878*, isoform A	NP_610733.1
Drosophila simulans	GD15248	XP_002076301.1
Drosophila sechellia	GM20384	XP_002033485.1
Drosophila erecta	GG22606	XP_001975983.1
Drosophila yakuba	GE13474	XP_002091126.1
Drosophila ananassae	GF12642	XP_001958964.1
Drosophila pseudoobscura pseudoobscura	GA21385	XP_001360093.2
Drosophila grimshawi	GH22153	XP_001987873.1
Drosophila willistoni	GK23220	XP_002074728.1
Drosophila mojavensis	GI19382	XP_002004832.1
Drosophila virilis	GJ22443	XP_002050972.1
Drosophila persimilis	GL11027	XP_002015431.1
Drosophila melanogaster	ballchen, isoform A	NP_651508.1
Homo sapiens	VRK1	NP_003375.1
Homo sapiens	VRK2	AAH21663.1
Mus musculus	VRK1, isoform A	NP_035835.1
Mus musculus	VRK2	AAH13520.1
Drosophila melanogaster (DNA sequence)	*CG8878* mutant 1a27a	KF287637
Drosophila melanogaster (DNA sequence)	*CG8878* mutant 3a22a	KF287638
Drosophila melanogaster (DNA sequence)	*CG8878* mutant 3a52a	KF287639
Drosophila melanogaster (DNA sequence)	*CG8878* mutant 3a66a	KF287640
Drosophila melanogaster (DNA sequence)	*CG8878* mutant 3a90a	KF287641

Table S1 Polypeptide accession numbers used. Note: the *D. persimilis* sequence was modified by the removal of one nucleotide from the DNA sequence to alter the amino acid reading frame to facilitate alignment.
